# Pathological Complete Response of Advanced Rectal Cancer Treated by Preoperative Chemoradiotherapy with Oral Tegafur-Uracil and Leucovorin: A Case Report

**DOI:** 10.1155/2013/175263

**Published:** 2013-11-30

**Authors:** Masaki Wakasugi, Toru Masuzawa, Mitsuyoshi Tei, Takeshi Omori, Shigeyuki Ueshima, Masayuki Tori, Masahiko Tsujimoto, Hiroki Akamatsu

**Affiliations:** ^1^Department of Surgery, Osaka Police Hospital, 10-31 Kitayama-cho, Tennoji-ku, Osaka 543-0035, Japan; ^2^Department of Pathology, Osaka Police Hospital, 10-31 Kitayama-cho, Tennoji-ku, Osaka 543-0035, Japan

## Abstract

A rare case of pathological complete response of advanced rectal cancer treated by preoperative chemoradiotherapy (CRT) with oral tegafur-uracil and leucovorin is reported. A 73-year-old man with bloody stool was diagnosed with type 2 rectal cancer located 6 cm from the anal verge. Examination of biopsy specimens revealed moderately differentiated adenocarcinoma. Computed tomography scans showed no distant or lymph node metastases. With a diagnosis of advanced lower rectal cancer of T3N0M0 stage III according to the TNM classification, he underwent preoperative CRT with oral tegafur-uracil and leucovorin. He did not experience any adverse events due to CRT. An abdominal CT scan and colonoscopy after CRT demonstrated significant tumor reduction. Then, 63 days after CRT, he underwent laparoscopic-assisted low anterior resection and diverting ileostomy. Pathological examination revealed no residual cancer cells. During 15 months of follow-up after his ileostomy was taken down, the patient continued to do well without any signs of recurrence or metastasis. Preoperative CRT with tegafur-uracil and leucovorin may thus represent a safe, well-tolerated, and effective therapeutic strategy for patients with advanced rectal cancer.

## 1. Introduction

Locally advanced rectal cancer is usually treated with preoperative chemoradiotherapy (CRT). After CRT and surgery, 8–33% of patients have no residual viable tumor on pathological examination, a pathological complete response (pCR) [1–4]. Oral tegafur-uracil (UFT) and leucovorin (LV) are safe and well tolerated anticancer drug therapies that do not decrease health-related quality of life (QOL) [5]. A case of pCR of advanced rectal cancer treated by preoperative CRT with oral UFT and LV is described.

## 2. Case Presentation

**Figure 1:**
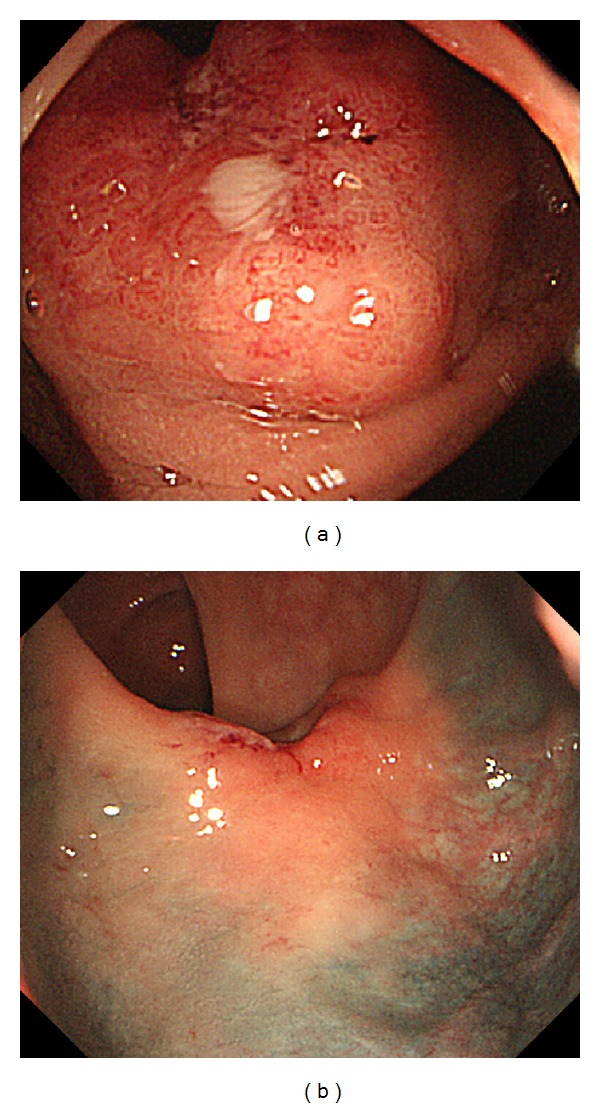
Colonoscopy before (a) and after (b) chemoradiotherapy (CRT).

**Figure 2:**
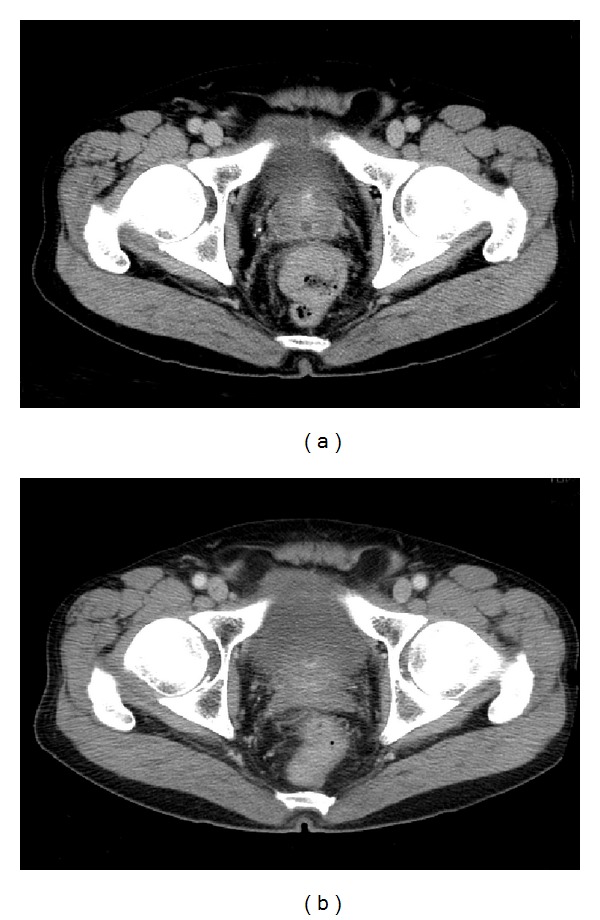
Abdominal computed tomography (CT) findings both (a) before and (b) after CRT.

**Figure 3:**
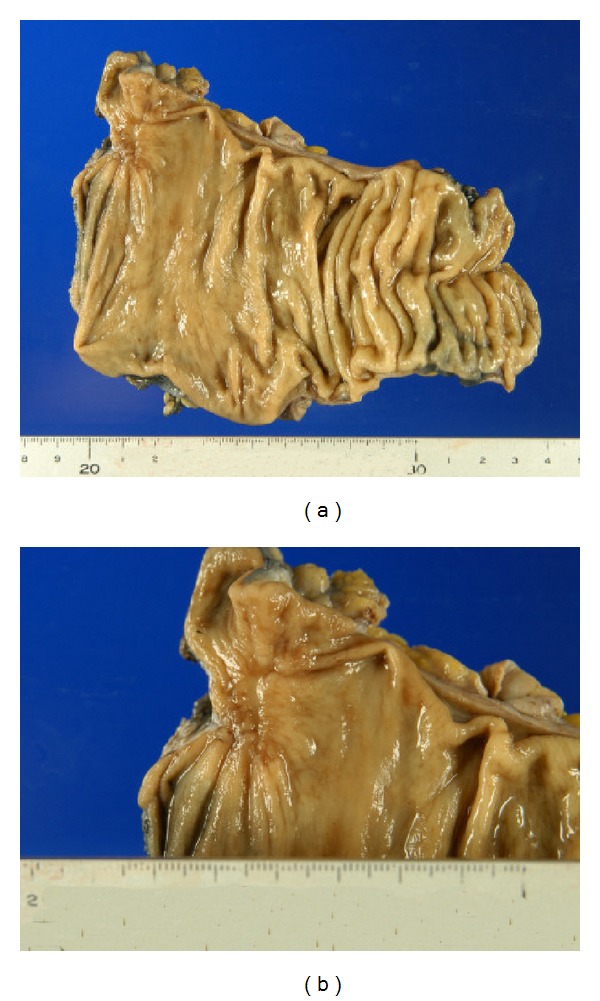
Resected specimen showing only ulcer scar.

**Figure 4:**
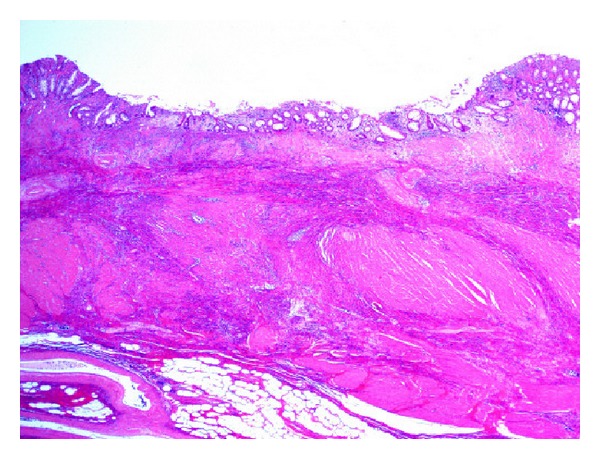
Pathological examination showing no tumor cells (hematoxylin-eosin stain).

A previously healthy 73-year-old man presented with blood in the stool in March 2012. Rectal examination revealed a hard tumor in the lower rectum. Colonoscopy showed a type 2 rectal tumor located 6 cm from the anal verge. Examination of biopsy specimens revealed moderately differentiated adenocarcinoma. The serum levels of carcinoembryonic antigen (CEA) and carbohydrate antigen 19-9 (CA19-9) were 1.5 ng/mL and 17 U/mL, respectively. Computed tomography (CT) scans showed no distant or lymph node metastases. These findings led to a diagnosis of advanced lower rectal cancer of T3N0M0 stage III according to the TNM classification. Therefore, preoperative CRT using oral UFT plus LV was performed before surgery. UFT/LV was administered orally at a dose of 500 mg/day and 75 mg/day, respectively, during the cycle (4 weeks on and 1 week off). During the cycle, radiotherapy was initiated concurrently, and a total dose of 50.4 Gy with 25 fractions of 1.8 Gy being given 5 days per week was given. The patient did not experience any adverse events due to CRT. An abdominal CT scan and colonoscopy performed after CRT demonstrated significant tumor reduction (Figures 1 and 2). On day 63 after CRT, in July 2012, laparoscopic-assisted low anterior resection and diverting ileostomy were performed. The surgical specimen of the rectum showed only ulcer scar (Figure 3). Pathological examination revealed no residual cancer cells and showed pCR (Figure 4). The patient's ileostomy was taken down 3 months after surgery. During the 15 months of follow-up, the patient continued to do well without any signs of recurrence or metastasis.

## 3. Discussion

The clinical course of this patient suggested two important clinical issues. First, preoperative CRT with UFT and LV has the potential to cure advanced rectal cancer. Second, preoperative CRT with oral UFT and LV is safe and well tolerated.

First, preoperative CRT with UFT and LV has the potential to cure advanced rectal cancer. In recent years, encouraging results with preoperative CRT have been reported. Wang et al. [6] reported that preoperative CRT with oral UFT and LV is effective, with tumor downstaging in 75% (39/52), pCR in 25% (13/52), and sphincter preservation in 55% (16/29) of lower-seated tumors, with tolerable toxicity in rectal cancer. Although few studies have addressed the survival benefit of preoperative CRT in patients with advanced rectal cancer [1, 2], it has been reported that pCR is obtained in 8% to 33% of patients receiving preoperative CRT [1–4] and that patients who achieve pCR show an improved oncologic outcome [3, 4]. Maas et al. [7] reported that 5-year crude disease-free survival was 83.3% (95% CI, 78.8–87.0) for patients with pCR (61/419 patients had disease recurrence) and 65.6% (63.6–68.0) for those without pCR (747/2263; HR 0.44, 95% CI, 0.34–0.57; *P* < 0.0001) and suggested that patients with pCR after CRT have better long-term outcomes than those without pCR.

Second, preoperative CRT with UFT and LV is safe and well tolerated. Many kinds of anticancer agents and molecular target drugs are used for preoperative CRT for rectal cancer. Among these, 5-fluorouracil (5-FU) is a key drug for preoperative CRT. The composition of UFT is 1-(2-tetrahydrofuryl)-5-fluorouracil (tegafur) and uracil in a molar ratio of 1 : 4. Tegafur is converted to 5-FU in vivo. The coadministration of uracil enhanced both the concentration of 5-FU in tumors and the resulting antitumor activity of tegafur [8]. Kopec et al. [5] compared health-related QOL, symptoms, and convenience of care in patients with colon cancer who received either oral UFT plus LV or standard intravenous fluorouracil plus LV as adjuvant chemotherapy, and they concluded that both regimens did not differ in health-related QOL and that the patients perceived adjuvant treatment with UFT and LV as more convenient than treatment with standard intravenous fluorouracil plus LV. In the present patient, preoperative CRT was completed without a severe impact on QOL, and this therapy contributed to sphincter preservation and curative resection. Preoperative CRT with UFT and LV was safe and well tolerated by the present patient, and it appears to have the potential to cure advanced rectal cancer without surgery.

In conclusion, preoperative CRT with tegafur-uracil and leucovorin appears safe and well tolerated, and it may be an effective therapeutic strategy for patients with advanced rectal cancer. More evaluation is required to confirm its efficacy and toxicity in a larger patient population.
